# Effects of open and closed skill exercise interventions on executive function in typical children: a meta-analysis

**DOI:** 10.1186/s40359-023-01317-w

**Published:** 2023-11-30

**Authors:** Xiaosu Feng, Ziyun Zhang, Teng Jin, Peng Shi

**Affiliations:** 1 School of Physical Education, Liaoning Normal University, Dalian, 116029 China; 2 School of Life and Health, Huzhou College, Huzhou, 313002 China; 3 School of Physical Education, Northeast Normal University, Changchun, 130024 China; 4 School of Physical Education, Shanghai University of Sport, Shanghai, 200438 China

**Keywords:** Sport skills, Executive function, Open skills, Closed skills, Typical children

## Abstract

**Background:**

The effects of open and closed skill exercise interventions for executive function in children and adolescents have received widespread attention. Open skill refers to the skill of performing motor tasks in an unpredictable environment; closed skill refers to the skill of performing motor tasks in a stable environment. However, the results of related studies are currently controversial and Meta-analysis is urgently needed.

**Methods:**

After computer searches of CNKI, Wan-Fang, VIP, WOS, PubMed, and EBSCO databases, two researchers independently screened articles, extracted information, and evaluated the quality of the articles. This study was statistical analyzed using Stata 16.0 software.

**Results:**

A total of 31 articles were included, including 2988 typical children. Open, closed, continuous and sequential skills all improved executive function in typical children to varying degrees, but open and sequential skills were more effective in improving executive function, particularly in the former in the working memory (*SMD*=-0.833, *P* < 0.001) and in the latter in the inhibitory control (*SMD*=-0.834, *P* < 0.001) and cognitive flexibility (*SMD*=-0.903, *P* < 0.001). Long-term, moderate- intensity interventions were better than acute, vigorous-intensity interventions for executive function, with long-term interventions reflected in working memory (*SMD*=-0.579, *P* < 0.001) and moderate-intensity interventions reflected in all three dimensions of executive function (*P* < 0.01). Intervention periods, intervention intensity and continuous and sequential skills classified by action structure play a significant moderating role. Better results for long-term, sequential structural action interventions based on open skills (*P* < 0.001); better results for acute, moderate intensity, sequential structural action interventions based on closed (*P* < 0.05). Whereas intervention intensity had a non-significant moderating effect in the open skills intervention, both moderate and vigorous intensity had a significant effect on executive function (*P* < 0.001).

**Conclusion:**

Open and closed skills have different levels of facilitation effects on executive function in typical children, but open skills are more effective. The facilitation effects of open and closed skills were moderated by the qualitative characteristics and action structure of the intervention.

## Introduction

Executive function is a higher cognitive function of the central nervous system that refers to the process of monitoring and controlling an individual’s thoughts and behaviors [1–3]. Executive function is generally considered to be a multidimensional structure consisting of three main components: inhibitory control, working memory and cognitive flexibility [4, 5], and has been validated and recognized by subsequent researchers [6, 7]. The childhood stage is a critical stage of cognitive development, and executive function at this stage is concerned with future academic performance as well as the formation and consolidation of creative awareness, health literacy, and good social relationships [8–11]. Therefore, how to promote children’s executive functions has become a topic of focus for researchers.

Currently, computer-operated tasks dominate executive function measurements. The researchers often use task paradigms such as Flanker, GO/NO GO, Stroop, and Stop signal to test inhibitory control; task paradigms such as N-back, Digit Span, and Sternberg to test working memory; and task paradigms such as More-Odd Shifting (MOS) and Task switching to test cognitive flexibility. The advantages of using computer-operated tasks are: first, these procedures save time in administering and scoring the test while ensuring the accuracy of data collection, and computer-based testing is more appropriate when the assessor is faced with practical constraints and limited time for subjects [12]; second, computer-operated tasks is consistent with the assumption that executive function is complex and multidimensional [13], which helps the researcher to explore the roles of the various subcomponents of executive functioning in the performance of the behaviors.

The relationship between physical exercise and executive function is receiving increasing attention. The results of the related systematic review and Meta-analysis showed that physical exercise promotes the improvement of executive function in children and adolescents, and that regular exercise of moderate intensity [14], more than three times per week, and with a single duration of 35 min or more has better benefits in promoting executive function in children and adolescents [15, 16]. Although the above systematic review and Meta-analysis confirmed the effectiveness of exercise interventions for executive function in children and adolescents, the included studies existed a large number of exercise interventions in laboratory settings, such as power bike and running table. And such interventions ignore the complexity of exercise in natural environments, so ecological validity is low and cannot be generalized to the real world [17]. Some researchers [18–20] have called for a focus on qualitative features of exercise interventions (e.g. metabolic energy supply, types of sport skills) and more real-world research to better facilitate translation of research findings.

Where sport skills are a combination of mental processes and skill manipulation processes [21], there are shared brain area mechanisms for skill learning and cognitive tasks. The cognitive benefits generated by exercise may be different in skill types [22], which may be related to the action tasks of the activities involved [23]. Depending on the predictability of the environmental context, sport skills can be divided into open and closed skills. The former refers to the skill of performing motor tasks in an unpredictable environment, where individuals need to react and adjust their movements to changes in the environment; the latter refers to the skill of performing motor tasks in a stable, predictable environment, where individuals are able to plan their movement routines in advance [24]. Within this conceptual framework, researchers [25–27] have found that participants with open skills outperformed participants with closed skills in some aspects of executive function; however, some studies [28, 29] have also reported no differences between the two.

There are several possible reasons for the above dispute. First, the intervention effects of open and closed skills on executive function may be moderated by the quantitative characteristics of exercise. Related studies [30, 31] demonstrated that moderate intensity was more beneficial to the development of executive function, while Chen et al. [32] compared the effects of different intensity basketball dribbling interventions and found that high intensity also helped to improve inhibitory control and working memory in school-aged children. The learning of complex sport skills improves the peer relationships of children and adolescents, makes it easier to stimulate enjoyment of exercise and positive emotional experiences, delays fatigue caused by exercise and helps to improve executive function [33]. Second, the effects of interventions in executive function by open and closed skills may be modulated by the structure of action. Depending on the complexity of the action structure, sport skills can be divided into sequential skills and continuous skills. The former refers to the joining of several discrete actions in a certain sequence to form a more complex action sequence; the latter is a multiple repetition of a single discrete action, and the action has no clear beginning and end, and the structure of the action is relatively single [24]. Shi et al. [27] found that sequential skills such as aerobics, which emphasize physical coordination [34], promoted executive function better than continuous skills such as running. In addition, this study found that skills with both open and sequential attributes had the best facilitation benefits for executive function. However, the systematic review by Shi et al. [27] was unable to calculate estimated effect sizes for exercise types, so the accuracy, stability and reliability of their results are questionable.

Based on this, this study was conducted by systematically searching for relevant studies based on Shi et al. [27]. This systematic review and meta-analysis had two research objectives. Firstly, a systematic review of research on the intervention of open and closed skills on typical children’s executive function is presented, and the effects of the interventions are compared quantitatively through Meta-analysis. Secondly, the moderating role of quantitative features of intervention and structural features of action in open and closed skills is explored. Through this study, it is hoped that it will inform subsequent research and teaching practice.

## Methods

This study was conducted in compliance with Preferred Reporting Items for Systematic Reviews and Meta-Analyses (PRISMA 2020) and was registered at International Prospective Register of Systematic Reviews (PROSPERO), under number CRD42023452385.

### Search strategies

A search of the relevant literature was conducted by one researcher using both English and Chinese search terms. Searches were conducted in the CKNI、Wan-Fang、VIP、Web of Science (WOS), PubMed and EBSCO databases using the following three sets of search terms. (1)“skill” “sports” “exercise” “fitness”; (2) “executive function” “working memory” “inhibition control” “cognitive flexibility” “self-control” “self-regulation”; (3) “children” “child” “pupil”. The search time frame is from the creation of this database to May 2022.

### Inclusion and exclusion criteria

Inclusion and exclusion criteria for the literature were designed according to the PICOS principles [35]. Inclusion criteria: (1) the participants were typical children under 14 years of age; (2) interventions are acute or long-term exercise interventions based on a variety of sport skills in real-world settings; (3) control measures include traditional physical education courses, basic academic courses, free movement or meditation, etc.; (4) outcome variables include inhibitory control, working memory, cognitive flexibility; (5) study designs include randomized controlled trials (RCT), randomized crossover designs (RCD) and quasi-experimental designs (QED). Exclusion criteria: (1) non-experimental studies; (2) reviews, abstracts, letters, comments; (3) type of sport skill not reported or not identified; (4) combined skills interventions for open and closed skills; (5) Screen-based physical games, such as Xbox, Kinect and Nintendo; (6) combined physical exercise and cognitive therapy intervention; (7) repeated publications on the same study subjects, including only relatively high quality literature; (8) raw data (mean and standard deviation) are not available. Selection out independently by two researchers and the selected literature was secondarily assessed by two other researchers and, if controversial, mutually agreed by group discussion.

### Data extraction

Extracts included first author, date of publication, study design, participants’ characteristics, interventions, controls and outcome variables, and the extracts were entered into Excel 2010 and saved. Three categories of evaluation indicators, namely response time, accuracy and score, were used to reflect the executive function of the subjects in the included studies, with faster response time, higher accuracy and higher score indicating better executive function. Therefore, accuracy and scores were back-calculated and extracted for coding in this study to maintain consistency with the direction of evaluation at the time of response, in preparation for the subsequent meta-analysis. The data extraction was carried out independently by two researchers and the extraction was secondarily assessed by two other researchers, and if there were controversial issues, a group discussion was held to decide jointly.

### Quality assessment

This study used the risk of bias assessment tool recommended by the Cochrane Collaboration Network to assess the risk of bias in randomized trials [36]. The tool is assessed from six aspects: randomization methods, blinding, allocation concealment, integrity of result data, selective reporting of study results and other biases. In this study, the MINORS scale [37] was used to assess the quality of the QED. The tool consists of 12 items, 9 to 12 of which are used to assess additional criteria for studies with a control group, each with a score of 2, for a total score of 24 [27]. A score of 0 means not reported; a score of 1 means reported but with insufficient information; a score of 2 means reported and sufficient information provided [27]. Judgments based on the assessment tool were made independently by two researchers and, where there was significant disagreement, the items were discussed with a third researcher.

### Statistical methods

This study uses Stata16.0 software for statistical analysis. Meta-analysis used standardized mean difference (*SMD*) for effect sizes and 95% confidence interval (*CI*) for the estimated intervals of the overall parameters constructed from the sample statistics. The *Q* test and *I*^*2*^ statistic were used to test for heterogeneity between included studies. If *I*^*2*^ < 50% and *P* > 0.1, heterogeneity was considered small and a fixed-effects model was selected for analysis; if *I*^*2*^ ≥ 50% and *P* ≤ 0.1, heterogeneity was considered large and a random-effects model was selected for analysis [38]. This study explores the moderating role of quantitative characteristics of the intervention and structural characteristics of the action through subgroup analysis. This study used Egger linear regression models for publication bias test. In this study, sensitivity analysis was carried out using the one-by-one elimination method and the cut-and-patch method. The level of heterogeneity was set at *α* = 0.1 and the rest of the tests at *α* = 0.05.

## Results

### Literature selection results

**Fig. 1:**
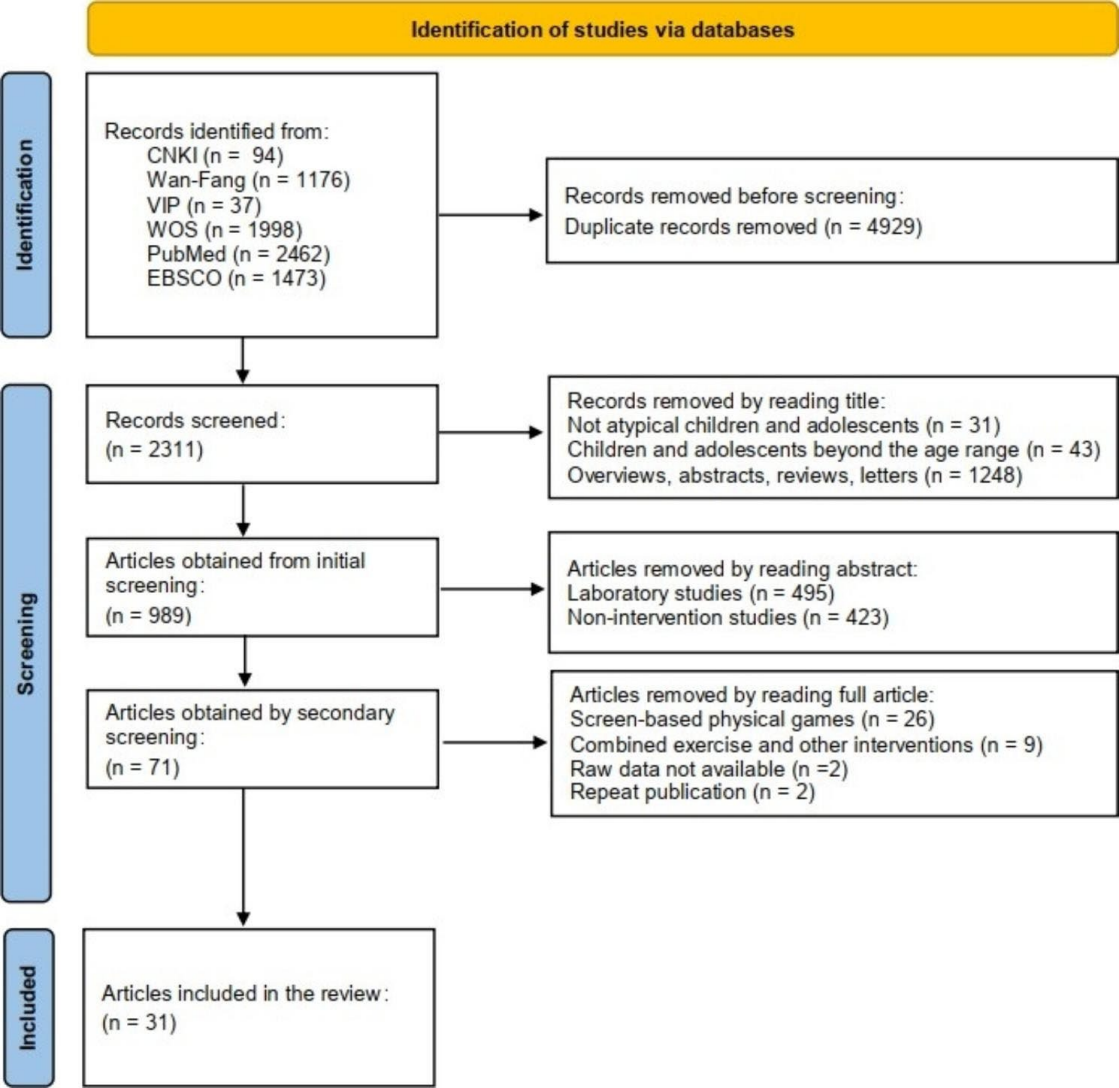
Flow chart for literature selection

A total of 7240 articles were retrieved, including 1307 Chinese articles and 5933 English articles. The retrieved articles were imported into EndNote X9 software for de-duplication, and 2311 articles were finally obtained. A total of 31 articles were included after literature selection. The literature selection process is shown in Fig. 1.

### Literature extraction results

The 31 articles included 12 (38.7%) acute intervention studies and 19 (61.3%) long-term intervention studies; included 25 (80.6%) RCTs, 2 (6.5%) RCDs and 4 (12.9%) QEDs. The 31 articles included 2988 typical children aged 3 to 13 years. 21 (67.7%) articles reported the proportion of girls among the subjects, with the proportion of girls ranging from 18.8 to 64.8% in all but 2 [39, 40] articles for boys only. 9 (75.0%) of the acute intervention articles reported the exercise intensity of the intervention, mostly moderate, with a single intervention duration of 10 to 50 min. 14 (73.7%) of the articles in the long-term intervention reported the intensity of the intervention, mostly moderate, and the quantitative characteristics of the remaining interventions were 6 to 36 weeks, 1 to 5 times/week and 30 to 120 times/min. Descriptive information on more participants, intervention and control measures, outcome variables measured and results are shown in Table 1.

**Table 1:** Basic characteristics of the included studies

Included studies	Study design	Participants’ characteristics (sample size / age / F%)		Interventions	Controls	Outcome variables		
		Test group	Control group			Inhibitory control	Working memory	Cognitive flexibility
Niemann et al. [41], 2013	RCT	27/9.7 ± 0.4y/NC	15/9.7 ± 0.5y/NC	12 min high intensity (85–90% HRmax) track and field running (II, III)	Sedentary	d2-test (T>C)	NC	NC
Palmer et al. [42], 2013	RCD	16/49.4 ± 5.3 m/18.8%	NC	30 min passing, dribbling and throwing activities (I, IV)	Sedentary	PDTP (T = C)	NC	NC
Yan et al. [34], 2014	RCT	T1 = 52/9.8 ± 0.3y/53.8% T2 = 51/9.7 ± 0.3y/49.0%	51/9.8 ± 0.3y/49.0%	30 min moderate intensity (60–69% HRmax) aerobics (II, IV) (T1) vs. obstacle run (I, III) (T2)	Sedentary	Flanker (T1>T2>C)	1-back (T1>T2>C)	More-odd shifting (T2>T1>C)
Chen et al. [32], 2014a	RCT	T1 = 30/9.8 ± 0.3y/50.0% T2 = 30/9.8 ± 0.3y/53.3% T3 = 32/9.7 ± 0.3y/46.9%	28/9.8 ± 0.3y/50.0%	30 min low intensity (50–59% HRmax) basketball dribbling (I, IV) (T1) vs. moderate intensity (60–69% HRmax) (T2) vs. vigorous intensity (70–79% HR max) (T3)	Free activities in their classroom	Flanker (T2>T1 = T3>C)	1-back (T2 = T3>T1 = C)	More-odd shifting (T2>T3 = C>T1)
Chen et al. [43], 2014b	RCT	44/3 ~ 5 g/47.7%	38/3 ~ 5 g/55.3%	30 min moderate intensity (60–70% HRmax) track and field group run (II, III)	Sedentary reading	Flanker (T>C)	2-back (T>C)	More-odd shifting (T>C)
Chen et al. [44], 2015a	RCT	T1 = 39/9.1 ± 0.3y/48.7% T2 = 38/9.1 ± 0.3y/44.7%	38/9.2 ± 0.4y/77.7%	30 min moderate intensity (60–69% HRmax) cooperative rope skipping (I, III) (T1) vs. single rope skipping (II, III)	Sedentary reading	Flanker (T1>T2>C)	1-back (T1>T2>C)	More-odd shifting (T1>T2>C)
Chen et al. [45], 2015b	RCT	24/9.5 ± 0.3y/NC	22/9.5 ± 0.3y/NC	30 min moderate intensity (60 ~ 69% HRmax) basketball dribbling (I, IV)	Free activities in the classroom	Flanker (T1>T2>C)	1-back (T1>T2>C)	More-odd shifting (T1>T2>C)
Jäger et al. [46], 2015	RCT	T1 = 54/134.6 ± 6.6 m/64.8% T2 = 62/135.3 ± 6.5 m/45.2%	58/135.8 ± 6.3 m/56.9%	20 min moderate intensity (70% HRmax) cognitive involvement skill games (I, IV) (T1) vs. aerobic exercise without cognitive involvement (II, III) (T2)	Sedentary exercise without cognitive involvement	Flanker (T1 = T2 = C)	1-back (T1 = T2 = C)	More-odd shifting (T1 = T2 = C)
Gallotta et al. [47], 2015	RCT	T1 = 31/8 ~ 11y/NC T2 = 46/8 ~ 11y/NC	39/8 ~ 11y/NC	50 min brisk walking, jogging, jumping (II, III) (T1) vs. basketball skills learning (I, IV) (T2)	Basic academic programme	d2-test (T2>T1>C)	NC	NC
Cooper et al. [48], 2016	RCD	12.6 ± 0.6/52.3%	NC	10 min high intensity interval sprint (II, III)	Sedentary	Stroop (T>C)	Corsi blocks test (T = C)	DSST (T = C)
Stein et al. [49], 2017	RCT	48/72.2 ± 5.2 m/50.0%	53/72.3 ± 6.9 m/52.8%	20 min motor skill learning based on coordination of both sides of the body (II, IV)	Board game	Simon-says task (T>C) Hearts-and-Flowers task- inconsistent tasks (T = C)	NC	Hearts-and-Flowers task- mixed tasks (T = C)
O’Brien et al. [39], 2021	RCT	T1 = 16/7.0 ± 0.5y/0.0% T2 = 16/6.7 ± 0.1y/0.0%	19/7.0 ± 0.5y/0.0%	30 min Open skills activities such as basketball, football, tennis (I, IV) (T1) vs. Closed skills activities such as racing, skipping (II, III) (T2)	Classroom activities	NC	Backward Digit Span (T1>T2>C) Corsi blocks test (T1 = T2 = C) Motor span task (T2>T1>C)	NC
Lakes et al. [50], 2004	RCT	207/kindergarten to grade five/NC		12 weeks martial arts (II, IV) intervention, 2–3 times/week, 45 min/time (T)	Traditional physical education course	NC	Digit Span (T1 = T2 = C)	NC
Chang et al. [51], 2013	QED	T1 = 13/7.2 ± 0.3/46.2% T2 = 13/7.0 ± 0.3/53.9%	NC	8 weeks low intensity (40–50% HRmax) football study (I, IV), 2 times/week, 35 min/time (T1) vs. moderate intensity (60–70% HRmax) (T2)	NC	Flanker (T1 = T2)	NC	NC
Lakes et al. [52], 2013	RCT	50/12.2y/52.00%	31/12.3y/48.00%	36 weeks taekwondo (I, IV), 2 times/ week, 45 min/time	Traditional physical education course	Hearts-and-Flowers task- inconsistent tasks (T = C)	NC	Hearts-and-Flowers task- mixed tasks (T = C)
Telles et al. [53], 2013	RCT	T1 = 49/10.4 ± 1.2y/30.6% T2 = 49/10.5 ± 1.3y/46.9%	NC	12 weeks yoga (II, IV), 5 times/week, 45 min/time (T1) vs. jogging, sprint running, relay races, bending, side bending, twisting (II, III)	NC	Stroop (T1>T2)	NC	NC
Crova et al. [54], 2014	RCT	37/9.6 ± 0.5y/46.0%	33/9.6 ± 0.5y/54.6%	21 weeks moderate intensity (150.5 ± 6.4 bpm) tennis (I, IV), 1 time/week, 120 min/time	Traditional physical education course	RNG-inhibition of average index (T>C)	RNG-updating of average index (T = C)	NC
Yin et al. [55], 2014	RCT	326/grade three to five/ 47.9%		20 weeks moderate intensity (120–140 bpm) martial arts + rope skipping + Fig. 8 running (II), 3 times/week, 30 min/time (T1) vs. figure running (I, III), 5 times/week (T2)	Blank control	Flanker (T1>T2 = C)	2-back (T2>T1>C)	More-odd shifting (T1 = T2>C)
Jiang et al. [56], 2015	RCT	31/5 ~ 6y/NC	30/5 ~ 6y/NC	8 weeks moderate intensity (60–70% HRmax) football games (I, IV), 2 times/week, 35 min/time	Blank control	Panda-Lion task (T>C) Snow-Green Grass task (T>C)	Corsi blocks test (T = C) Reverse Corsi blocks test (T = C)	Flexible project selection task (T = C)
Schmidt et al. [57], 2015	RCT	T1 = 69/11.3 ± 0.6y/62.3% T2 = 57/11.3 ± 0.6y/50.9%	55/11.4 ± 0.6y/49.1%	6 weeks vigorous intensity soft hockey and basketball games (I, IV), 2 times/week, 45 min/time (T1) vs. 200 m round trip run (II, III) (T2)	Traditional physical education course	Flanker (T1>T2 = C)	2-back (T1>T2 = C)	More-odd shifting (T1>T2 = C)
Chen et al. [58], 2016	RCT	20/11.4 ± 0.6y/NC	20/11.3 ± 06y/NC	8 weeks moderate intensity (60–69% HRmax) physical and mental exercise exercises (II, IV), 3 times/week, 40 min/time	Regular academic life	Flanker (T = C)	1-back (T>C)	More-odd shifting (T>C)
Pan et al. [59], 2016	RCT	25/12.0 ± 0.6y/NC	23/12.0 ± 0.6y/NC	10 weeks moderate intensity (60–69% HRmax) basketball intervention (I, IV), 3 times/week, 30 min/time	Traditional physical education course	Flanker (T>C)	1-back (T>C)	More-odd shifting (T>C)
Alesi et al. [40], 2016	QED	24/8.8 ± 1.1y/0.0%	20/9.3 ± 0.9y/0.0%	24-week football intervention (I, IV), 2 times/week, 75 min/time	Traditional physical education course	NC	Forward Digit Span (T = C) Backward Digit Span (T = C) Corsi blocks test (T>C)	NC
Pesce et al. [25], 2016	RCT	232/5 ~ 10y/50.4%	228/5 ~ 10y/49.6%	24 weeks moderate intensity (131.9 ± 17.4 bpm) motor coordination and cognitive engagement based skill games (I, IV), 1 time/week, 60 min/time	Traditional physical education course	RNG-inhibition of average index (T>C)	RNG-updating of average index (T = C)	NC
Chen et al. [60], 2017	RCT	21/9.4 ± 0.5y/47.6%	20/9.2 ± 0.4y/50.0%	8 weeks moderate intensity (60–69% HRmax) football intervention (I, IV), 2 times/week, 40 min/time	Traditional physical education course	Flanker (T>C)	1-back (T>C)	More-odd shifting (T>C)
Yin et al. [61], 2017	RCT	26/4 g/NC	21/4 g/NC	16 weeks moderate intensity (60–69% HRmax) basketball intervention (I, IV), 3 times/week, 30 min/time	Blank control	Flanker (T>C)	1-back (T>C)	More-odd shifting (T>C)
Cho et al. [62], 2017	RCT	15/11.2 ± 0.8y/40.0%	15/11.3 ± 0.7y/40.0%	16 weeks moderate intensity (RPE = 11–15) taekwondo intervention (I, IV), 5 times/week, 60 min/time	Blank control	Stroop (T>C)	NC	NC
Dai [63], 2020	QED	46/10.5 ± 0.3y/NC	43/10.4 ± 0.3y/NC	24 weeks moderate intensity (60–69% HRmax) football intervention (I, IV), 5 times/week, 120 min/time	Blank control	Flanker (T>C)	2-back (T>C)	Salthouse (T>C)
Lai et al. [64], 2020	RCT	10/5 ~ 7y/50.0%	10/5 ~ 7y/50.0%	8 weeks moderate intensity (60–69% HRmax) tennis intervention (I, IV), 2 times/week, 60 min/time	Basic academic course	NC	1-back (T>C)	NC
Oppici et al. [65], 2020	RCT	T1 = 30/8.8 ± 0.5y/62.0% T2 = 30/8.7 ± 0.7y/59.0%	20/8.9 ± 0.7y/63.0%	7 weeks high cognitive dance exercise, 2 times/week, 60 min/time (II, IV) (T1) vs. low cognitive dance exercise (II, IV) (T2)	Blank control	Flanker (T = C)	List Sorting Working Memory test (T = C)	Dimensional Change Card Sort test (T = C)
Ma et al. [66], 2022	QED	40/9.2 ± 0.3y/NC	40/9.2 ± 0.3y/NC	16 weeks football intervention (I, IV), 3 times/week, 40 min/time	Blank control	GO/NO GO (T>C)	1-back (T>C) 2-back (T>C)	More-odd shifting (T>C)
Abbreviations and notes: T = Test group; C = Control group; y = year; m = month; F%= Percentage of girls; NC = Not clear; I = open skills; II = closed skills; III = continuous skills; IV = sequential skills; HRmax = maximum heart rate; bpm = beats per minute of the heart; RPE = Rating of Perceived Exertion; PDTP = Picture Deletion Task for Preschoolers; DSST = Digit Symbol Substitution Test; RNG = Random Number Generation task.								

### Risk of bias assessment results

**Fig. 2:**
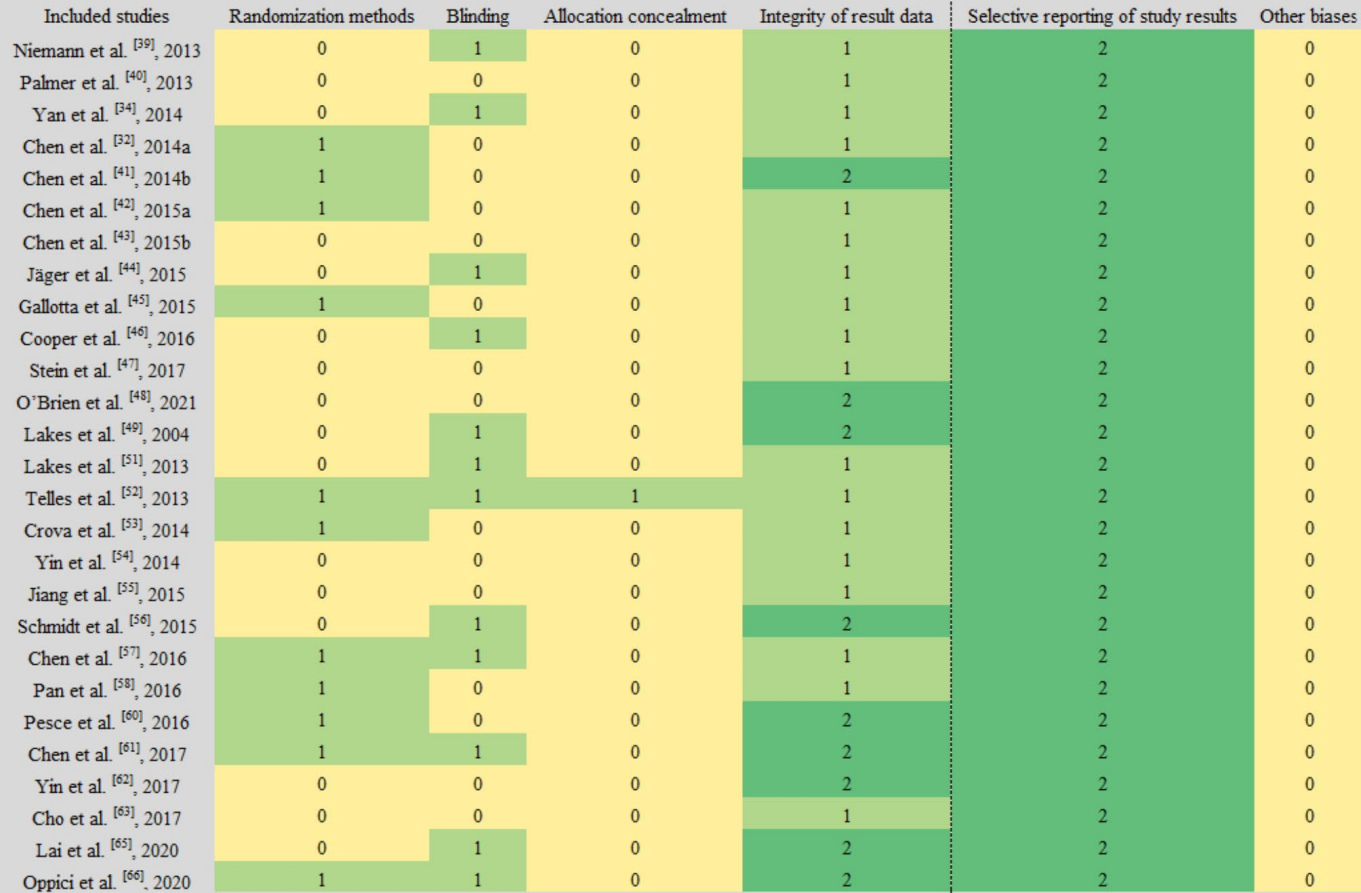
Results of risk of bias assessment for RCT and RCD studies (Notes: 0 means “not clear”, 1 means “yes” and 2 means “no”.)

**Fig. 3:**
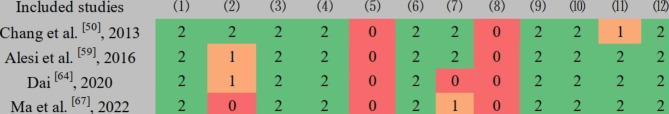
Results of risk of bias assessment for QED studies (Notes: 0 means “not reported”, 1 means “reported but with insufficient information”, 2 means “reported and sufficient information provided”)

Of the 27 randomized controlled trials (RCTs and RCDs), 11 (40.7%) studies reported randomization methods; 12 (44.4%) studies reported strategies for administering blinding, only one study [53] reported strategies for allocation concealment; 16 (59.3%) studies reported completeness of outcome data and no subject dropouts/ missed visits; all studies were free from bias in selective reporting of study results and it was unclear whether other biases existed (Fig. 2). The results of the quality assessment of the 4 QEDs showed that most entries were reported and provided detailed and informative information, resulting in an overall low likelihood of bias (Fig. 3).

### Meta-analysis results

#### Comparison of sport skill types

There was a large heterogeneity between included studies (*I*^*2*^ > 50%, *P* < 0.1), so a random effects model was chosen for analysis. The results of the combined effects test (Table 2) showed that both open and closed skills had a significant improvement in inhibitory control and cognitive flexibility in typical children (*P* < 0.05), while open skills (*SMD*=-0.833, *P* < 0.001) were better than closed skills (*SMD*=-0.539, *P* = 0.088) for improving working memory; continuous skills (*SMD*=-1.124, *P* = 0.003) and sequential skills (*SMD*=-0.903, *P* < 0.001) had a significant improvement in cognitive flexibility for typical children, while sequential skills (*P* < 0.001) were better than continuous skills for both inhibitory control and working memory (*P* > 0.05).

**Table 2:** Results of a test of the combined effect of different types of sport skill exercise on executive function interventions

Categories	Outcome variables	Heterogeneity test		Combined effects test			
		*I* ^*2*^	*P*	*SMD*	95%*CI*	*Z*	*P*
Open skills	Inhibitory control	91.8%	< 0.001	-0.942	(-1.290, -0.594)	5.30	< 0.001
	Working memory	93.0%	< 0.001	-0.833	(-1.220, -0.447)	4.22	< 0.001
	Cognitive flexibility	94.1%	< 0.001	-1.116	(-1.658, -0.575)	4.04	< 0.001
Closed skills	Inhibitory control	94.9%	< 0.001	-0.524	(-0.957, -0.091)	2.37	0.018
	Working memory	96.5%	< 0.001	-0.539	(-1.158, 0.080)	1.71	0.088
	Cognitive flexibility	94.8%	< 0.001	-0.854	(-1.353, -0.356)	3.36	0.001
Continuous skills	Inhibitory control	97.2%	< 0.001	-0.450	(-1.203, 0.303)	1.17	0.242
	Working memory	97.6%	< 0.001	-0.048	(-1.002, 0.905)	0.10	0.921
	Cognitive flexibility	96.3%	< 0.001	-1.124	(-1.863, -0.384)	2.98	0.003
Sequential skills	Inhibitory control	88.3%	< 0.001	-0.834	(-1.090, -0.578)	6.39	< 0.001
	Working memory	92.0%	< 0.001	-0.889	(-1.224, -0.554)	5.20	< 0.001
	Cognitive flexibility	94.1%	< 0.001	-0.903	(-1.375, -0.403)	3.76	< 0.001

#### Comparison of quantitative intervention characteristics

Only one study [32] explored the effect sizes of low-intensity exercise interventions for working memory and cognitive flexibility, and it was not possible to calculate heterogeneity. Due to the large heterogeneity among the remaining included studies (*I*^*2*^ > 50%, *P* < 0.1), a random effects model was chosen for the analysis. The results of the combined effects test (Table 3) showed that both the acute and long-term interventions were significantly (*P* < 0.01) effective in improving inhibitory control and cognitive flexibility in typical children, while the long-term intervention (*SMD*=-0.579, *P* < 0.001) was better than the acute intervention (*SMD*=-0.753, *P* = 0.056) in improving working memory; The moderate and low intensity (*P* < 0.01) interventions were more effective than the vigorous intensity (*P* > 0.05) intervention in improving inhibitory control and working memory in typical children, and the moderate intensity intervention (*SMD*=-1.394, *P* < 0.001) was more effective than the vigorous intensity (*SMD*=-0.992, *P* = 0.158) and low intensity (*SMD* = 0.312, *P* = 0.238).

**Table 3:** Intervention effects of different quantitative characteristics on executive function

Quantitative characteristics	Categories	Outcome variables	Heterogeneity test		Combined effects test			
			*I* ^*2*^	*P*	*SMD*	95%*CI*	*Z*	*P*
Cycle	Acute	Inhibitory control	95.9%	< 0.001	-0.886	(-1.400, -0.332)	3.18	0.001
		Working memory	97.0%	< 0.001	-0.753	(-1.524, 0.019)	1.91	0.056
		Cognitive flexibility	96.0%	< 0.001	-0.717	(-1.165, -0.269)	4.00	< 0.001
	Long-term	Inhibitory control	82.9%	< 0.001	-0.612	(-0.890, -0.334)	4.31	< 0.001
		Working memory	89.7%	< 0.001	-0.579	(-0.870, -0.288)	3.90	< 0.001
		Cognitive flexibility	92.9%	< 0.001	-0.075	(-1.524, 0.019)	3.14	0.002
Intensity	Low	Inhibitory control	86.4%	< 0.001	-1.827	(-3.113, -0.540)	2.78	0.005
		Working memory	—	—	-1.649	(-2.248, -1.050)	5.40	< 0.001
		Cognitive flexibility	—	—	0.312	(-0.206, 0.831)	1.18	0.238
	Moderate	Inhibitory control	95.3%	< 0.001	-0.851	(-1.304, -0.399)	3.69	< 0.001
		Working memory	96.3%	< 0.001	-0.892	(-1.433, -0.351)	3.23	0.001
		Cognitive flexibility	94.1%	< 0.001	-1.394	(-1.867, -0.920)	5.77	< 0.001
	Vigorous	Inhibitory control	94.5%	< 0.001	-0.421	(-1.218, 0.375)	1.04	0.300
		Working memory	98.0%	< 0.001	-1.382	(-3.046, 0.281)	1.63	0.103
		Cognitive flexibility	97.3%	< 0.001	-0.992	(-2.367, 0.384)	1.41	0.158

#### The moderating role of quantitative intervention characteristics

Given the limitations of the number of studies on low-intensity interventions, only the moderating effects of moderate and vigorous intensities will be explored. Due to the large heterogeneity among the included studies (*I*^*2*^ > 50%, *P* < 0.1), a random effects model was chosen for the analysis. The results of the combined effects test (Table 4) showed that both acute and long-term interventions based on open skills had a positive intervention effect on inhibitory control and cognitive flexibility in typical children (*P* < 0.05), while the long-term intervention (*SMD*=-0.730, *P* < 0.001) had a better intervention effect on working memory than the acute intervention (*SMD*=-1.167, *P* = 0.074); both acute and long-term interventions based on closed skills were not significant for working memory in typical children (*P* > 0.05), while acute interventions (*P* < 0.05) were better than long-term interventions for inhibitory control and cognitive flexibility (*P* > 0.05); moderate and vigorous intensity open skills interventions had significant intervention effects on inhibitory control, working memory and cognitive flexibility in typical children (*P* < 0.001); moderate and vigorous intensity closed skills interventions were not significant for inhibitory control and working memory in typical children (*P* > 0.05), while moderate intensity interventions (*SMD*=-1.562, *P* < 0.001) were better than vigorous intensity (*SMD*=-0.596, *P* = 0.391) for cognitive flexibility. In summary, the intervention cycle plays a moderating role in open skills interventions for working memory and in closed skills interventions for inhibitory control and cognitive flexibility; the intervention intensity exerts a moderating effect in closed skills intervention for cognitive flexibility, while the moderating effect in the open skills intervention is not significant.

**Table 4:** Results of tests of the moderating effect of quantitative intervention characteristics

Type of skills	Quantitative characteristics	Categories	Outcome variables	Heterogeneity test		Combined effects test			
				*I* ^*2*^	*P*	*SMD*	95%*CI*	*Z*	*P*
Open skills	Cycle	Acute	Inhibitory control	96.7%	< 0.001	-1.693	(-2.993,-0.393)	2.55	0.011
			Working memory	96.6%	< 0.001	-1.167	(-2.445,0.112)	1.79	0.074
			Cognitive flexibility	97.8%	< 0.001	-1.975	(-3.941,-0.008)	1.97	0.049
		Long-term	Inhibitory control	88.0%	< 0.001	-0.751	(-1.076,-0.427)	4.54	< 0.001
			Working memory	89.9%	< 0.001	-0.730	(-1.096,-0.365)	3.92	< 0.001
			Cognitive flexibility	92.3%	< 0.001	-0.899	(-1.439,-0.359)	3.26	0.001
Closed skills	Cycle	Acute	Inhibitory control	95.8%	< 0.001	-0.617	(-1.219,-0.015)	2.01	0.045
			Working memory	97.3%	< 0.001	-0.506	(-1.517,0.505)	0.89	0.372
			Cognitive flexibility	95.5%	< 0.001	-1.092	(-1.746,-0.438)	3.27	0.001
		Long-term	Inhibitory control	90.4%	< 0.001	-0.276	(-0.806,0.253)	1.02	0.306
			Working memory	88.4%	< 0.001	-0.221	(-0.708,0.265)	0.98	0.326
			Cognitive flexibility	93.9%	< 0.001	-0.329	(-1.163,0.506)	0.77	0.440
Open skills	Intensity	Moderate	Inhibitory control	93.1%	< 0.001	-0.983	(-1.465,-0.500)	3.99	< 0.001
			Working memory	94.8%	< 0.001	-1.110	(-1.705,-0.514)	3.65	< 0.001
			Cognitive flexibility	92.1%	< 0.001	-1.248	(-1.853,-0.642)	4.04	< 0.001
		Vigorous	Inhibitory control	95.2%	< 0.001	-1.382	(-1.777,-0.988)	6.87	< 0.001
			Working memory	92.1%	< 0.001	-1.975	(-2.407,-1.542)	8.95	< 0.001
			Cognitive flexibility	88.6%	< 0.001	-2.149	(-2.594,-1.704)	9.46	< 0.001
Closed skills	Intensity	Moderate	Inhibitory control	93.3%	< 0.001	-0.587	(-1.556,0.382)	1.19	0.235
			Working memory	97.4%	< 0.001	-0.328	(-1.400,0.744)	0.60	0.548
			Cognitive flexibility	95.7%	< 0.001	-1.562	(-2.331,-0.793)	3.98	< 0.001
		Vigorous	Inhibitory control	93.7%	< 0.001	-0.224	(-1.039,0.591)	0.54	0.590
			Working memory	97.8%	< 0.001	-1.184	(-3.154,0.786)	1.18	0.239
			Cognitive flexibility	96.1%	< 0.001	-0.596	(-1.956,0.765)	0.86	0.391

#### The moderating role of continuous and sequential skills

Due to the large heterogeneity among the included studies (*I*^*2*^ > 50%, *P* < 0.1), a random effects model was chosen for the analysis. The results of the combined effects test (Table 5) showed that both open-continuous and open-sequential skills had significant intervention effects on working memory and cognitive flexibility in typical children (*P* < 0.05), while open-sequential skills (*SMD*=-0.825, *P* < 0.001) had a better intervention effect on inhibitory control than open-continuous skills (*SMD*=-1.883, *P* = 0.071); closed-sequential skills (*P* < 0.01) were more effective than closed-continuous skills (*P* > 0.05) in intervening with inhibitory control, working memory and cognitive flexibility in typical children. In summary, continuous and sequential skills play a moderating role in open skill intervention inhibitory control and a moderating role in closed skill intervention executive function.

**Table 5:** Results of tests of the moderating effect of continuous and sequential skills

Environmental context	Action structure	Outcome variables	Heterogeneity test		Combined effects test			
			*I* ^*2*^	*P*	*SMD*	95%*CI*	*Z*	*P*
Open skills	Continuous skills	Inhibitory control	98.4%	< 0.001	-1.883	(-3.928, 0.162)	1.80	0.071
		Working memory	98.1%	< 0.001	-1.886	(-3.766, -0.006)	1.97	0.049
		Cognitive flexibility	95.9%	< 0.001	-2.314	(-3.650, -0.978)	3.40	0.001
	Sequential skills	Inhibitory control	85.7%	< 0.001	-0.825	(-1.122, -0.528)	5.44	< 0.001
		Working memory	89.2%	< 0.001	-0.668	(-1.020, -0.317)	3.72	< 0.001
		Cognitive flexibility	92.8%	< 0.001	-0.790	(-1.369, -0.211)	2.67	0.007
Closed skills	Continuous skills	Inhibitory control	97.0%	< 0.001	-0.032	(-0.910, 0.846)	0.07	0.943
		Working memory	97.6%	< 0.001	0.672	(-0.533, 1.876)	1.09	0.274
		Cognitive flexibility	94.5%	< 0.001	-0.537	(-1.289, 0.216)	1.40	0.162
	Sequential skills	Inhibitory control	91.1%	< 0.001	-0.864	(-1.350, -0.378)	3.48	< 0.001
		Working memory	95.1%	< 0.001	-1.385	(-2.190, -0.581)	3.38	0.001
		Cognitive flexibility	95.3%	< 0.001	-1.054	(-1.845, -0.263)	2.61	0.009

### Tests for publication bias

The Egger linear regression model constructs a linear regression equation with the effect size as the dependent variable and the precision of the effect estimate as the independent variable. The intercept of the regression equation is the bias, and the closer it is to 0, the less likely there is publication bias, and if *P* > 0.05 and the 95% *CI* contains 0, and then there is no publication bias [38]. The results in Table 6 show that *P* > 0.05 and 95% *CI* contains 0 in inhibitory control and working memory, indicating that there was no publication bias in the included studies and that the Meta-analysis results were stable and reliable; while *P* < 0.05 and 95% *CI* contains 0 in cognitive flexibility, suggesting a possible publication bias in the included studies.

**Table 6:** Results of Egger linear regression analysis

Outcome variables	*β*	*SE*	*t*	*P*	95%*CI*
Inhibitory control	-2.118	1.600	-1.32	0.192	(-5.345, 1.107)
Working memory	-2.033	1.681	-1.21	0.234	(-5.436, 1.370)
Cognitive flexibility	-5.675	2.445	-2.32	0.027	(-10.675, -0.675)

### Sensitivity analysis

In this study, sensitivity analysis was carried out with the help of the “metaninf” command for the one-by-one rejection method. For inhibitory control, *SMD*=-0.859 to -0.653, 95% *CI*=(-1.104 to -0.914, -0.614 to -0.393) after excluding one study at a time. For working memory, *SMD* = -0.802 to -0.655, 95% *CI* = (-1.144 to -0.984, -0.609 to -0.326) after excluding one study at a time. For cognitive flexibility, *SMD* = -1.034 to -0.866, 95% *CI* = (-1.403 to -1.219, -0.664 to -0.512) after excluding one study at a time. None of the results of the combined effects tests after excluding one study at a time changed substantially. In addition, this study used the cut-and-patch method proposed by Duval et al. [67] to identify and correct for funnel plot asymmetries caused by publication bias. The results showed four new studies added after the cut-and-patch method, with a combined effect size *SMD* = 0.553, 95% *CI* = (0.369, 0.829). As a result, there was no significant change from the combined effect size before the cut-and-patch, and the results are robust and reliable [68].

## Discussion

### Effects of open and closed skills exercise interventions on executive function

Both open and closed skills contribute to improved inhibitory control and cognitive flexibility in typical children, but open skills is far more effective interventions for working memory than closed skills. Open and closed skills have different degrees of facilitative benefits on executive function, but given the different effects of different types of sport skills on brain organization and neural activation, there are differences in the outward expression of executive control they produce. Sustained closed skills exercise promotes cerebral neovascularization, increases cerebral blood flow, and activates inhibitory control-related brain areas [69, 70]. However, open skills exercise that combines environmental enrichment, interpersonal interaction, and motor coordination is more likely to promote neurogenesis and synaptic neogenesis, and promote increased activation in brain regions associated with attention control and working memory [17, 69, 71]. In addition, regular participation in open skills exercise over a long period of time will also combine the advantages of closed skills exercise and therefore be more effective in promoting the executive function [72, 73]. In addition, cognitive flexibility is one of the more complex skills in executive function, with all conscious attentional control and transfer dependent on the development of inhibitory control and working memory and their coordination with each other [74]. Whereas the results suggest that closed skills are less effective in improving working memory, they have good facilitative benefits for cognitive flexibility. This may stem from the moderating effect of structural characteristics of action. Complex coordinated body movements are more conducive to mobilizing the storage and processing functions of the working memory system in order to flexibly complete the transition between thought and movement [34]. In conclusion, open skills are more effective interventions for executive function in typical children.

### Effects of quantitative intervention characteristics on executive function

Long-term interventions are more effective in executive function, particularly in the working memory dimension. The longer the intervention period the better the facilitation effect is demonstrated and the long-term intervention effect is much higher than the effect of the 1-time intervention [75]. Acute interventions can increase activation levels in the dorsolateral prefrontal cortex and increase cerebral blood flow; whereas long-term interventions can increase structural plasticity in brain grey and white matter and improve functional brain networks, so the latter has a higher intervention effect and follow-up effect [27]. In particular, improvements in working memory are based on functional connections between the default mode network and the frontal, posterior and temporal cortices in the executive control network [76] and therefore require prolonged intervention to achieve.

The results were similar to those of previous studies on dose-effect relationships [32, 77], in that moderate intensity interventions were more effective overall on executive function. Although the low-intensity intervention had good intervention effects on inhibitory control and working memory, it is difficult to draw conclusions given the limitations of the number of included studies. Meanwhile, empirical studies [32, 51] have shown that low-intensity interventions can improve some subcomponents of executive function compared to controls, but the magnitude of improvement is not as great as for moderate-intensity interventions. Vigorous intensity interventions are least effective because the self-control power model assumes that there is a finite amount of energy available for self-control. Energy expended on previous self-control that is not restored in time may lead to ego depletion, which will affect subsequent self-control behaviors [78]. Therefore, this study supports the idea of the self-control strength model.

### Moderating effect of quantitative intervention characteristics

Intervention cycles moderate the effects of both open and closed skills interventions, with long-term open skills being more effective for working memory and acute closed skills being more effective for inhibitory control and cognitive flexibility. Previous studies have shown that open skills and long-term interventions in particular have better intervention effects on working memory in typical children, mainly due to the need for open skills to control perceptual-motor coordination and physical-motor coordination [77, 79], as well as the increased brain plasticity and functional connectivity of executive control networks that result from long-term interventions [76, 80]. In addition, closed skills are more effective in intervening with inhibitory control and cognitive flexibility, and sustained exercise increases brain arousal levels and blood flow and activates networks related to motor control [79, 81]. Sequential combinations of limb movements increase the cognitive demand on the brain and increase dynamic activation of the frontoparietal network to improve fixation switching functions [34]. In closed skills, acute interventions are more effective than long-term interventions, mainly stemming from the fact that individuals gradually adapt to existing environmental stimuli during long-term movements and that individuals are not faced with new problems and challenges, which may therefore lead to stagnation or even a slight decline in the development of executive functions [82]. Sport skills learning are cognitive and associative in nature. Individuals inhibit irrational visuomotor planning in the early stages and assess new visual stimuli and kinesthetic information through working memory refreshes in order to flexibly complete stereotypic shifts in thought and movement, activating specific prefrontal areas [83]. However, as sport skills reach an automatic level, activation in prefrontal areas decreases and the role of consciousness in the control of individual movements is minimized.

Intervention intensity had a non-significant moderating effect in open skills and a moderating effect in closed skills, where moderate intensity interventions were better for cognitive flexibility than vigorous intensity. The results of this study showed that both moderate- and vigorous-intensity open-skill exercises significantly improved the executive function of typical children. Organized open skills learning and competitions such as football and basketball are more likely to increase motivation to exercise, increase positive emotional experiences, build and strengthen peer bonds, and delay fatigue from exercise [33, 84]. Therefore, this may provide some offset to the negative benefits of vigorous intensity exercise on executive function and does not strictly satisfy the hypothesis of an inverted U-shaped relationship. However, it is important to note that only two [32, 57] papers have explored the effects of vigorous intensity open-skill interventions, which need to be further tested in subsequent studies. However, for closed skills, the findings support the hypothesis of an inverted U-shaped relationship between intervention intensity and executive function. The original inverted-U hypothesis was derived from a laboratory environment task based on a power bike or running Table [85]. Movement in this environment lacks interpersonal interaction and rich environmental stimuli and is therefore similar to closed skills.

### Moderating effects of continuous and sequential skills

Both continuous and sequential skills contribute to improved cognitive flexibility in typical children, but sequential skills are much more effective in improving inhibitory control and working memory than continuous skills. Sequential skills have a complex movement structure and are movement sequences that combine motor coordination and aerobic fitness. The involvement of multiple limbs and the flexibility of movements during the task require more mental manipulation processes to be involved [20]. The motor process requires the brain to give rapid operational instructions depending on external stimuli (e.g. musical rhythms), to suppress information that has been activated but is not relevant to the target action, to correct musical and motor instructions in working memory, and to be flexible to complete the transition between thought and action, more easily increasing blood flow to the dorsolateral prefrontal cortex [50, 86]. Sequential skills are therefore more effective as an intervention than continuous activities alone.

Continuous and sequential skills play a moderating role in both open and closed skill interventions, and skills with sequential structural movement properties are more effective in promoting executive function in typical children. Specifically, open-sequential skills intervened better than open-continuous skills for inhibitory control; closed-sequential skills intervened better than closed-continuous skills for inhibitory control, working memory and cognitive flexibility. A recent Meta-analysis [87] evaluated the effect of 11 sport skills on working memory in school-age children, with a general pattern of “open > closed, sequential > continuous”.

### Limitations

The following limitations of this study remain. Firstly, the search process is limited by language, which may lead to publication bias. However, sensitivity analyses of the one-by-one elimination and cut-and-patch methods showed robust and reliable results, and the findings were similar to those of similar published studies [87, 88]. Secondly, there is a risk of bias in the methodological quality of the included articles, which may confound the results of the intervention to some extent. Finally, the paucity of literature on the executive function of low-intensity interventions, limited by primary sources, makes it difficult to draw valid conclusions and to test for subsequent moderating effects.

## Conclusion

The aim of this study was to quantitatively compare the effects of open and closed skills interventions on executive function in typical children, as well as to explore the moderating role of quantitative intervention characteristics and movement structure characteristics in open and closed skills interventions. The results of the combined effects test of the 31 papers showed that overall interventions for open skills exercise were better than closed skills; sequential skills were better than continuous skills; and long-term interventions, moderate intensity, were better than acute interventions, vigorous intensity. Moderating effects showed better interventions for long-term open skills and better interventions for acute closed skills; Moderate intensity interventions are more effective than vigorous intensity; intervention effects were better for open-sequence skills than for open-continuous skills and for closed-sequential skills than for closed-continuous skills. Intervention practices should design interventions based on the personality characteristics of the subject and select the type of exercise that interests them in order to better promote improved executive functioning in children and adolescents.

## Data Availability

The data that support the findings of this study are available on request from the corresponding author upon reasonable request.
